# Imaging plant cell death: GFP-Nit1 aggregation marks an early step of wound and herbicide induced cell death

**DOI:** 10.1186/1471-2229-5-4

**Published:** 2005-03-29

**Authors:** Sean R Cutler, Chris R Somerville

**Affiliations:** 1 Department of Botany, University of Toronto, 25 Willcocks St., Toronto, Ontario, M5S 3B2, Canada; 2 Carnegie Institution, Department of Plant Biology, 260 Panama Street, Stanford, CA 94305, USA

## Abstract

**Background:**

A great deal is known about the morphological endpoints of plant cell death, but relatively little is known about its sequence of events and / or its execution at the biochemical level. Live cell imaging using GFP-tagged markers is a powerful way to provide dynamic portraits of a cellular process that can in turn provide a descriptive foundation valuable for future biochemical and genetic investigations.

**Results:**

While characterizing a collection of random GFP-protein fusion markers we discovered that mechanical wounding induces rapid aggregation of a GFP-Nitrilase 1 fusion protein in Arabidopsis cells directly abutting wound sites. Time-lapse imaging of this response shows that the aggregation occurs in cells that subsequently die 30 – 60 minutes post-wounding, indicating that GFP-Nit1 aggregation is an early marker of cell death at wound sites. Time-lapse confocal imaging was used to characterize wound-induced cell death using GFP-Nit1 and markers of the nucleus and endoplasmic reticulum. These analyses provide dynamic portraits of well-known death-associated responses such as nuclear contraction and cellular collapse and reveal novel features such as nuclear envelope separation, ER vesiculation and loss of nuclear-lumen contents. As a parallel system for imaging cell death, we developed a chemical method for rapidly triggering cell death using the herbicides bromoxynil or chloroxynil which cause rapid GFP-Nit1 aggregation, loss of nuclear contents and cellular collapse, but not nuclear contraction, separating this response from others during plant cell death.

**Conclusion:**

Our observations place aggregation of Nitrilase 1 as one of the earliest events associated with wound and herbicide-induced cell death and highlight several novel cellular events that occur as plant cells die. Our data create a detailed descriptive framework for future investigations of plant cell death and provide new tools for both its cellular and biochemical analysis.

## Background

Events that compromise the integrity of a single cell can threaten the well-being of an entire multi-cellular organism, a fact reflected by the diverse mechanisms that organisms use to eliminate potentially dangerous cells [1]. For example, when injured beyond repair by high doses of irradiation or by treatment with cytotoxic agents, regulated cell death may be activated to remove damaged cells. This process is best understood in animal cells from extensive analyses of the mechanisms that underpin apoptosis, a regulated process marked by a set of stereotyped changes in cellular architecture that culminate in cell death [2,3].

In plant cells, regulated cell death occurs in numerous contexts, such as during development of xylem elements [4] or as part of the hypersensitive response (HR) to pathogen attack [5-7]. Although the hypersensitive response is triggered by highly specific plant-pathogen interactions and is under genetic control [7-9], the HR exhibits many similarities to plant responses triggered by wounding. Both processes activate localized mechanisms for forming protective barriers through lignification, cross-linking of cell wall proteins and other modifications of the extracellular matrix [10,11], which are thought to limit pathogen access. The HR and wound response display extensive overlaps in their transcriptional responses [12] and pathogen-derived elicitors of the HR can activate wound-induced kinase activities [13] suggesting that the two responses share some regulatory components. Thus, both wound and hypersensitive responses activate related defenses local to and transmitted from their primary sites of initiation, but differ in their activating signals.

There has been intensive research on the signaling mechanisms that regulate the myriad responses elicited by wounds and pathogens. Several lines of evidence suggest that the localized and transmitted components of both responses are regulated, in part, by hydrogen peroxide-mediated signaling events [14-17]. It has been proposed that H_2_0_2 _is a broad spectrum signaling molecule that triggers local processes like cell wall protein cross linking [14] and cell death [6] as well as long distance effects such as gene induction [11]. Although the precise mechanisms by which H_2_0_2 _signals are initiated locally and then transmitted are incompletely known, they involve activation of an NADPH oxidase that generates H_2_0_2 _via superoxide production [18], analogous to the oxidative defenses mounted by macrophages of the mammalian immune system. NO signaling also appears to participate in this oxidative response [19,20].

In contrast to the signal-mediated events that trigger these responses, relatively little is known about the downstream events that execute the orderly patterns of cellular deconstruction that accompany cell death. In animal cells many of the characteristic events are attributed to the activity of caspase proteases, which initiate and execute a cascade of proteolytic events that participate in subcellular deconstruction [21]. Parallels between plant cell death and animal apoptosis have been suggested from observations of cellular contraction, nuclear contraction and fragmentation of DNA during HR cell death. Plants have a family of caspase-related proteins, designated as metacaspases, and numerous studies have implicated caspase-like proteases in the control of cell death activation in plants [22]. Recently, a vacuolar protease unrelated to caspases at the amino acid sequence level has been found to posess caspase protease activity and has been demonstrated to be required for viral induced hypersentive cell death in tobacco [23], showing that caspase activities mediate some components of plant cell death. Nonetheless, the targets of this and other caspases that mediate the changes in subcellular architecture during plant cell death still remain elusive.

In the course of a screen for useful GFP fusion proteins [24], we observed that a GFP-Nitrilase 1 fusion protein exhibited a change of aggregation state in response to wounding. In order to place this phenomenon in a larger context, we initiated a series of live-cell imaging experiments to identify and characterize markers associated with the execution of physically and chemically induced cell death. Our studies reveal a series of previously undescribed events associated with wound-induced cell death in plants and raise new mechanistic questions regarding the execution of plant cell death.

## Results

### GFP-Nit1 aggregation marks an early step of wound induced cell death

**Figure 1:**
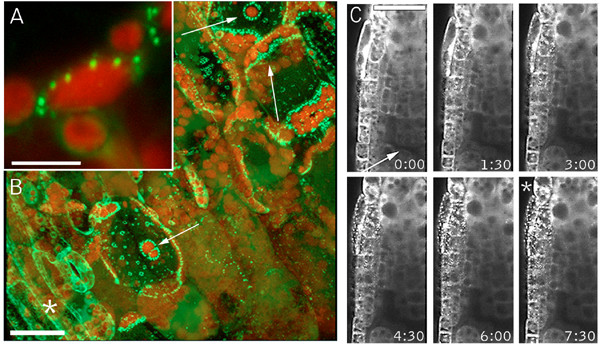
**Localized and transmitted wound responses of the GFP-Nit1 marker**. (A, B) Aggregation in cells abutting a wound site. A 35S GFP-Nit1 leaf petiole was severed by a razor blade incision at a 45° angle to the main axis of the petiole. Three mesophyll cells exposed by the wound are indicated by the white arrows (panel B) – note the extensive redistribution of the marker in these cells to organelle-associated aggregates, which is particularly evident around chloroplasts (red fluorescence). (C) Transmitted wound response. A 35S GFP-Nit1 seedling was mounted in 0.5X MS and punctured above the root meristem (wound site marked with an arrow). Imaging was initiated after an ~45 – 60 second delay between the wound and alignment of the root tip with field of view. Aggregates become evident several cell layers removed from the wound site (marked with an asterisk). Times shown are in minutes. The image shown in A and B are 3-dimensional reconstruction of a Z-series taken 5 min after wounding. Chloroplasts are visible because of their red autofluorescence. Images in C are single optical sections. Scale bars: A 10 μm; B, C 25 μm.

Our investigations of cell death arose from the discovery of a wound responsive GFP marker, GFP-Nit1, which was identified in a previous random marker screen [24]. The GFP-Nit1 construct encodes the full length coding sequence for the enzyme nitrilase 1 fused to the C-terminus of the coding sequence for GFP. In non-wounded cells, the fusion protein was present throughout the cytoplasm and nucleoplasm (comparable to soluble GFP) and was visible as diffuse fluorescence (results not presented). Following a puncture wound, GFP-Nit1 rapidly translocates from the cytosol to organelle-associated aggregates in cells adjacent to (Fig. 1A,B) and at a distance from wounds (Fig. 1C, and see additional file 1), suggesting that the marker responds to wound induced signals. Based on observations described below, we consider the local response at the wound site (defined here as the cells directly abutting cells damaged by the wound) as distinct from the transmitted response, which can spread many cell layers from the wound site.

**Figure 2:**
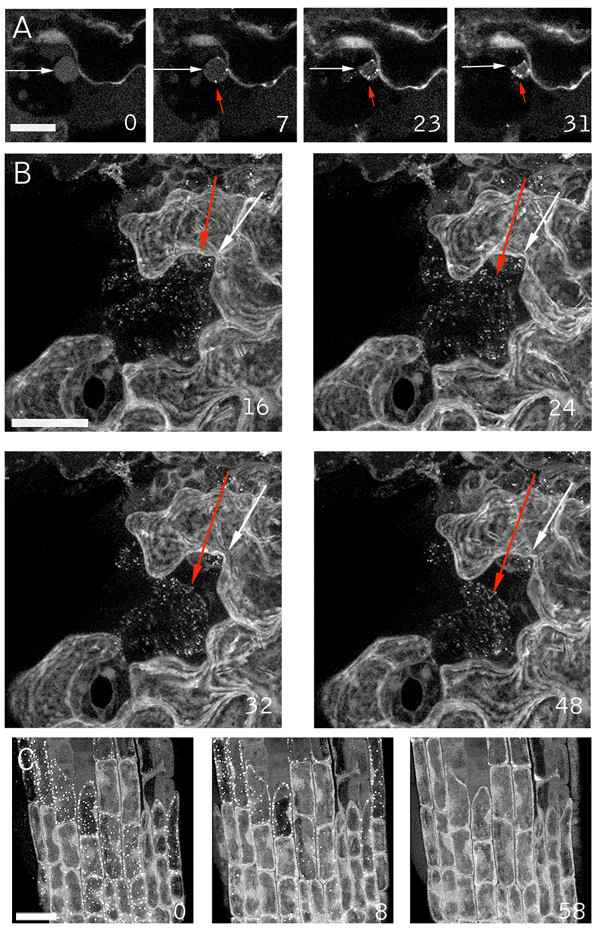
**Nuclear and cellular collapse during the wound response**. The GFP-Nit1 line N1P2E was wounded and a confocal Z-series was immediately collected at 60 sec intervals from a region abutting the wound. (A) Nuclear contraction of a nucleus (adjacent to the white arrow) in a wound-proximal cotyledon epidermal cell that after wounding displayed the aggregation response (aggregates indicated by red arrows) then collapsed. (B) Cellular collapse by one of several cells in the wound site that displayed GFP-Nit1 aggregates and contractions. The red arrow points to the edge of the contracting cell and the white arrow to the wall of an adjacent cell. Note the detachment of the cell from the wall (most evident at 48 min after wounding). (C) Reversibility of transmitted response. An agarose-mounted 35S GFP-Nit1 plant was wounded above the root meristem and imaged by the acquisition of Z-series at 2 min intervals for 60 min (see supplemental data for complete image series). In the first time point after wounding, the aggregation response had spread throughout much of the root meristem. Over time, the aggregates reverted back to a cytoplasmic distribution pattern and cytoplasmic streaming was evident. Images in A are single confocal optical sections, B and C are reconstructions from Z-series stacks. The numbers in the lower right of each panel indicate time in min from the start of imaging. Scale bars A, B = 25 μm; C = 20 μm.

To characterize the local response, we imaged cells in the direct vicinity of puncture wounds administered to hypocotyls or cotyledon tissue of transgenic GFP-Nit1 plants. We observed that a variable number of cells in the wound site displayed GFP-Nit1 aggregates and rounded nuclei (Fig. 2A). Time lapse imaging of several wound-zones demonstrated that after forming GFP-Nit1 aggregates, these cells displayed nuclear contraction (Fig. 2A) followed by dramatic cellular collapse (Fig. 2B and see additional file2) (n = 6 independent wound experiments). These two responses (cellular collapse and nuclear contraction) are morphological hallmarks of cell death during the HR [11,25,27], and suggested that the local GFP-Nit1 response marks one component of a more general wound-triggered cell death response. Unlike the local response, we found that the transmitted response was reversible, with cytoplasmic streaming resuming concomitant with disappearance of GFP-Nit1 aggregates (see additional file 3). Thus, our observations suggest GFP-Nit1 aggregation does not mark a point of commitment to cell death in all cells. It does however mark an early stage of a distinct cell death process that operates in cells abutting the wound site.

**Figure 3:**
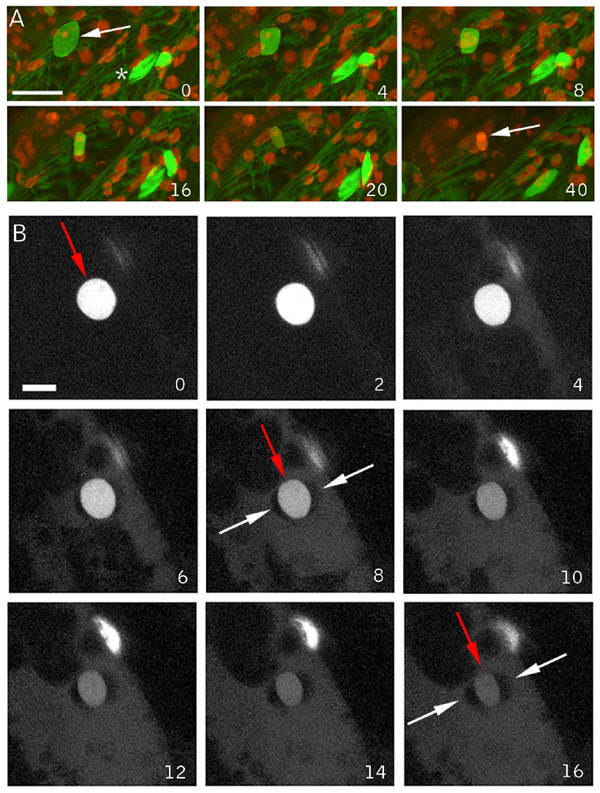
**Wound induced nuclear collapse in nuclear marker lines**. (A) Nuclear response in line N6. Following wounding of a hypocotyl, the nucleus (arrow) first swells then contracts. The asterisk at time = 0 illustrates the normal lentoid shape of hypocotyl nuclei. Contraction is followed by a decrease in GFP fluorescence, most evident between the 16 and 20-min time points. This is followed later by intense nuclear propidium iodide staining (shown in red, adjacent to arrow). (B) Nuclear response in a wounded hypocotyl of an N7 line. Contraction of the nucleus (red arrow), decrease in nuclear fluorescence and release of nuclear fluorescence into the cytoplasm are evident. Note the formation of lobes (marked with a white arrow) around the contracting nucleus (red arrow). The time series in A and B are brightest-point reconstructions. Times shown are in min. Scale bars: A = 25 μm, B = 10 μm.

To explore the possibility that the GFP-Nit1 fusion protein might induce an aberrant cell-death response, we examined whether the process occurred independently of the GFP-Nit1 fusion protein. We examined the nuclear contraction observed with GFP-Nit1 during the wound response using a previously isolated nuclear marker (N6) [24]. Hypocotyls of N6 GFP transgenic plants were wounded and cells adjacent to wounds continuously imaged over a one-hour period (Fig. 3). As observed with GFP-Nit1, a subset of cells in the wound site possessed swollen, hypertrophic nuclei that progressed to a contracted state (Fig. 3A and see additional file 4). Propidium iodide was included in the imaging buffer to monitor plasma membrane integrity during the response. Intense staining of nuclei occurred subsequent to the major nuclear contraction, suggesting that degeneration of plasma membrane integrity is a late step. Thus nuclear contraction during wound induced cell death is not an artifact induced by the GFP-Nit1 fusion protein, a conclusion supported by results with another nuclear marker and markers of the ER (see below). In addition, as noted below, we have developed a biochemical assay to probe Nit1 relocalization aggregation and find that this occurs in wild type plants (i.e. in the complete absence of any GFP fusion protein).

### Nuclear leakage and lobing accompany nuclear contractions

In addition to nuclear contraction, a wound-induced decrease in the intensity of GFP nuclear fluorescence in the N6 marker line was evident within less than 20 min of wounding (Fig. 3A). Several explanations for the decrease in nuclear fluorescence seemed possible, including GFP destruction, alterations in pH that quenched fluorescence, or the expulsion of nuclear contents into the cytoplasm. While these possibilities are not mutually exclusive, the latter seemed particularly attractive since contraction might cause extrusion of nuclear contents. The N6 marker possesses a low background level of cytoplasmic GFP localization that might mask the release of nuclear contents and also may be partially immobilized from the bulk nucleoplasm by an association with DNA (this marker illuminates chromosomes during mitosis, [26]). Consequently, we examined wound responses using the nuclear marker line N7, which displays a lower level of background cytoplasmic fluorescence and does not appear to be associated with chromatin as assessed by time-lapse imaging of mitoses (data not shown). Hypocotyls of plants carrying the N7 marker were wounded and imaged. Time-lapse imaging of cells proximal to wound sites revealed that a gradual illumination of the cytoplasm occurs concomitantly with the decrease in nuclear fluorescence and nuclear contraction, suggesting that the nuclear label is lost or expelled into the cytoplasm (Fig. 3B). The simultaneous illumination of nucleoplasm and cytoplasm facilitated visualization of the formation of lobes on the nuclei, revealed by negative contrast, as the interior of these lobes excludes the GFP label (See arrows in Fig. 3).

### Lobing is caused by nuclear envelope separation

**Figure 4:**
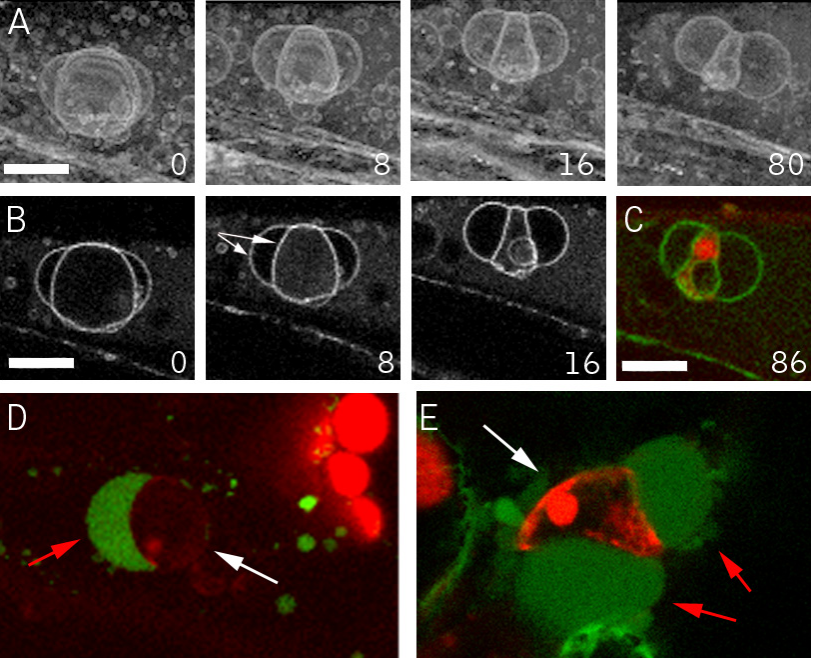
**Nuclear lobes are bound by the ER membrane and contain ER lumen contents**. A hypocotyl of the ER membrane marker line Q4 was wounded and imaged at 2-minute intervals by confocal microscopy. The simultaneous contraction and lobing of the nucleus are evident as a separation of the nuclear envelope. (A) 3D reconstruction of the contracting nucleus made with a brightest-point reconstruction of the acquired data set. (B) The same data set as in Fig. 4A at a single optical section through the mid-plane of the contracting nucleus, illuminating the double membrane structure (pointed to by arrows) and its separation. (C) A single optical section through the contracted nucleus shows propidium iodide staining (shown in red) of the interior, demonstrating that the nuclear lumen is interior to the lobes. (D, E) Hypocotyls of a line expressing the ER-lumenal GFP marker mGFP5 were wounded. Shown are contracting epidermal nuclei (white arrows) from two independent wound experiments using mGFP5 approximately 10 min after wounding. The inter-lobal space contains mGFP5 label (red arrows), indicating that this compartment is contiguous with the lumen of the ER. GFP fluorescence is shown in green and propidium iodide in red. Scale bars = 10 μm.

Since the nucleus is bound by the nuclear envelope, a membrane system contiguous with the ER (for review of the plant ER see [28]) lobing could conceivably occur if the inner membrane of the nuclear envelope contracted without a corresponding contraction of the outer membrane – i.e. if the two membranes separated. Exclusion of cytosolic GFP from the interior of the lobes might be expected if this were true, since the space between the inner and outer nuclear envelopes, the peri-nuclear space, is contiguous with the lumen of the ER and thus isolated from the cytoplasm. This hypothesis is supported by observations of two ER markers, Q4 an ER membrane marker and mGFP5, a KDEL-tagged lumenal ER marker. Figure 4 displays time points from a time-lapse wound imaging experiment using the Q4 marker line. Figure 4A shows three-dimensional reconstructions of one image series, and illustrates the characteristic lobes that form during nuclear contraction. Consistent with the hypothesis of nuclear envelope separation, optical sections through the mid-plane of the same contracting nucleus shown in 4A reveal a clear double membrane structure that separates concomitantly with nuclear contraction (Fig. 4B). We next tested the behavior of an ER-lumenal GFP marker (mGFP5) during nuclear contraction and lobing and found that nuclei at differing stages of contraction and lobing contain the ER-lumenal marker mGFP5 within the inter-lobal space (Fig. 4D,E).

Based on our observations that the inter-lobal space excludes cytosolic GFP, contains the lumenal ER marker mGFP5, and that the lobes are demarcated by a membrane system contiguous with the ER, we believe that the simplest explanation for the observed nuclear lobing is that the inner and outer membranes of the nuclear envelope separate during nuclear contraction. Thus, nuclear contraction does not reflect a contraction of the entire nucleus proper, but rather a contraction of the nuclear lumen and its tightly associated inner envelope.

### Degeneration of the cortical-ER network accompanies cell death

**Figure 5:**
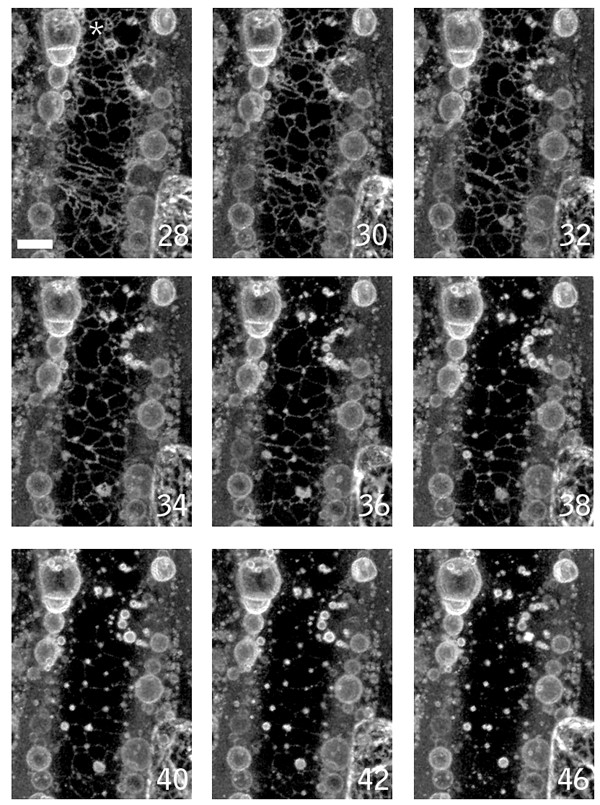
**ER vesiculation during wound induced cell death**. A wound-proximal petiole epidermal cell of ER membrane marker line Q4 was imaged. The panel shows time points (in min) after nuclear contraction. Prior to the times shown, part of the ER network formed bubble-like structures rapidly after wounding. The remaining intact tubular ER subsequently degenerated, forming large numbers of vesicular structures. Images are brightest point reconstructions of Z-series collected at 2 minutes intervals after wounding. Times shown are in minutes. Scale bar = 10 μm.

Both the ER tubules and the nuclear envelope contain closely appressed double-membrane structures, assemble with similar properties *in vitro *[28,43], and are contiguous membrane systems. Thus, the death response might be expected to additionally alter cortical ER tubules. To examine this, we imaged wound-proximal cells of the Q4 ER-membrane marker line (Fig. 5 and Additional file 5). About 20 min after wounding, extensive nuclear contraction and lobing was evident (marked with an asterisk in Fig. 5), part of the ER membrane system had formed bubble-like structures but much of the ER-tubular network was still intact. Shortly thereafter, the tubular ER network rapidly degenerated, contracting from tubules into small vesicular structures (Fig. 5). Thus, alterations in the integrity of both the nuclear envelope and cortical ER tubules occur during the local wound-induced cell death response, although apparently with different kinetics. Prior to ER tubule vesiculation, but concurrent with nuclear contraction and lobing, we observed that plastids in the cell moved away from the cell cortex (data not shown). Since plastid movements are dependent on a well-characterized actin-based motility system [29] these observations suggest that this system is at least partly intact during the earlier stages of wound induced cell death. Further investigation of this awaits imaging experiments using cytoskeletal markers.

### Benzonitrile herbicides induce rapid cell death

**Figure 6:**
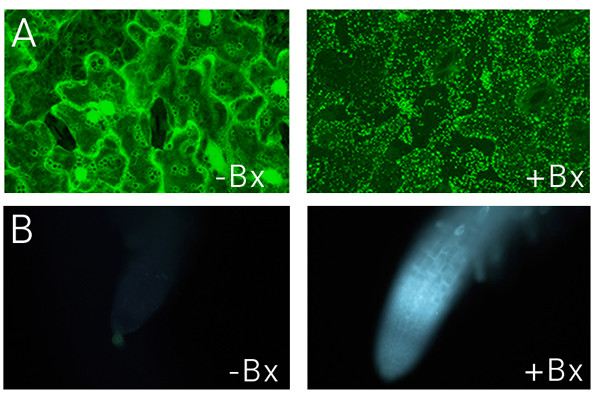
**GFP-Nit1 aggregation and callose induction by bromoxynil**. (A) 8 day old GFP-Nit1 plants (N1P2E) seedlings were imbibed in 500 μM Bromoxynil (+Bx) or mock solutions (-Bx) for 1 h then imaged by confocal microscopy, note the exstensive accumulation of the fusion protein in aggregates after herbicide treatment. (B) Callose accumulation following bromoxynil treatment. Wild type seedlings were treated with 500 μm bromoxynil (+Bx) or a mock-solution (-Bx). Roots were examined at regular intervals under UV illumination using conventional epifluorescence and a DAPI filter set; the 15 min time point is shown. Callose deposition was visualized using the callose specific stain sirofluor, added at 100 ug/ml at the beginning of the experiment. Sirofluor fluorescence was first visible approximately 8 min after addition of bromoxynil. Images were acquired on a digital camera using identical exposure settings.

To probe the specificity of the local wound responses we sought a simple method for chemically inducing cell death on a time frame similar to that used for our wounding experiments (45 – 90 min). Many chemically-induced forms of cell death have been characterized, but typically their effects operate on the time scale of hours to days as opposed to minutes. To identify a fast acting molecule we used the GFP-Nit1 aggregation response to screen a number of herbicides and other small-molecules for rapid activity, focusing on nitriles, which have been used to trigger cell death in other experiments (i.e., KCN, [5]). Potassium cyanide, while obviously toxic, did not induce the rapid GFP-Nit1 aggregation, cell death and cellular collapse on a 1 hour time scale that we sought [30]. We found that the hydroxy-benzonitrile herbicide bromoxynil (2,4 di-bromo-para-hydroxybenzonitrile), and its analog chloroxynil (2,4 di-chloro-para-hydroxybenzonitrile), caused rapid cell death when applied to Arabidopsis tissue at concentrations between 250 – 500 μM. Within 1 hour of application to seedlings bromoxynil and chloroxynil induced rapid GFP-Nit1 aggregation (Fig. 6A), extensive cell shrinkage and cell death (additional file 6 ). Like wounding, these herbicides also induced callose deposition (Fig. 6B), which was evident as early as 8 min after application to roots. The specific mode of action of these herbicides is unclear but they have well documented effects on mitochondrial respiration [31,32] and also bind to photosystem II proteins [33]. Whatever their mechanism of action, they proved convenient rapid inducers of woundless cell death for use in our imaging experiments.

### Nuclear and cellular contraction can be uncoupled during cell death

**Figure 7:**
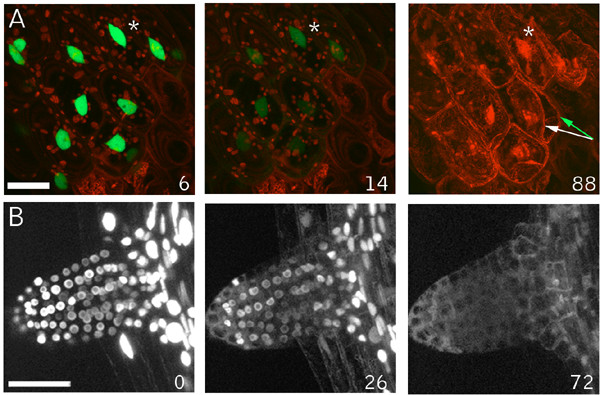
**Chloroxynil induced death – collapse of cells without nuclear contractions**. (A) The nuclear marker line N7 was mounted in agarose, and the bromoxynil-analog chloroxynil was added to a final concentration of 500 μM and a Z-series of the hypocotyl-root junction was collected at 2 min intervals. Loss of nuclear label into the cytoplasm and a generalized loss of GFP fluorescence were followed by staining of nuclei by propidium iodide and massive cellular collapse. The shape of the nuclei remained constant throughout the experiment and did not display the characteristic contractions seen during wound induced cell death. The white asterisk identifies the same nucleus, and the green arrows to two cells in this plane of view that displayed massive plasmolysis. (B) Chloroxynil induced release of N7 marker from nucleoplasm in 12 day old N7 plants mounted in agarose and overlaid with chloroxynil to a final concentration of 500 μM. An initiating lateral root was imaged by the acquisition of Z-series at 120 sec intervals. A gradual release of nuclear label and illumination of the cytoplasm occurs without the characteristic nuclear contractions induced by wounding. Scale bars = 25 μm. Inset numbers indicate min after herbicide addition.

To probe nuclear dynamics during chemically-induced cell death we examined hypocotyl cells of the N7 marker line after chloroxynil application. Time lapse imaging experiments showed that prior to the late stage inductions of cellular collapse and propidium iodide staining of nuclei, the N7 label leaves the nuclei, illuminating the cytoplasm analogously to wound induced cell death (Fig. 7B). However the characteristic lentoid shape of the hypocotyl nuclei persisted throughout, demonstrating that this hallmark of wound induced cell death does not occur during this particular type of chemically induced cell death (Fig. 7A). Notable in these experiments was the rapid loss of the GFP fluorescence, which was not seen to the same extent in our wounding experiments. We next examined the response in root cells, which also failed to show nuclear contraction but clearly show nuclear leakage of the GFP marker (Fig. 7B and additional file 6). Thus, in both cell types, nuclear leakage occurs without concomitant contractions of the nucleus, showing that these two responses can be separated. Importantly, this observation suggests that the simple hypothesis that nuclear leakage is caused by contraction-induced extrusion is unlikely.

### Fusiform-body spherulation accompanies wounding and chemical cell death

**Figure 8:**
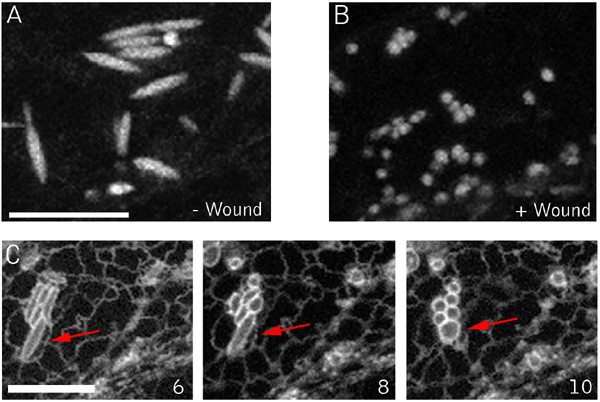
**ER Fusiform body alterations during wound and chloroxynil induced cell death**. (A) Hypocotyl cell of an unwounded plant showing the normal morphology of the fusiform bodies. (B) Wound proximal hypocotyl cell. Note the spherical bodies in this cell, distinct from the normally cigar-shaped structures illuminated by lumenal ER markers. (C) Chloroxynil-induced transitions. Epidermal hypocotyl cells were treated with 500 μM chloroxynil. Inset numbers indicate time (in minutes) after herbicide addition. An individual fusiform body throughout the experiment is marked by the red arrow. By 4 min after herbicide application, streaming ceases, evident by the lack of movement of fusiform bodies and the tubular ER network, which are usually highly dynamic. Shortly thereafter, fusiform bodies transform into spherical structures. Scale bar = 10 μm.

Markers of the ER in Arabidopsis illuminate a cigar-shaped accessory organelle called the fusiform body [34,35]. Recent evidence suggests that this organelle may be involved in cell death, but its specific functions are still unclear [36]. This structure is evident in Arabidopsis plants expressing the ER markers mGFP5 and Q4 (Fig. 8A). In our time-lapse observations of ER tubule and nuclear envelope degeneration using the Q4 marker line, the fusiform bodies were noticeably absent. This prompted us to examine them more closely using mGFP5, which brightly illuminates these structures [37,35]. In wound-proximal mGFP5-expressing cells, the most evident structures from the first time points after wounding are spheres, with fusiform bodies absent (Fig. 8A). This observation suggests that the fusiform bodies rapidly lose their characteristic morphology in response to wounding. To examine if this response occurs during herbicide-induced death, we imaged the Q4 ER-membrane marker line after application of chloroxynil. Shortly after addition of chloroxynil, cytoplasmic streaming stopped and this was quickly followed by the transformation of fusiform bodies into spherical structures (Fig. 8B,C and additional file 7). Thus, both wounding and herbicide treatment act at an early stage to effect component(s) required to maintain the distinctive shape of this organelle.

### GFP-Nit1 aggregates translocate into a pellet fraction during cell death

**Figure 9:**
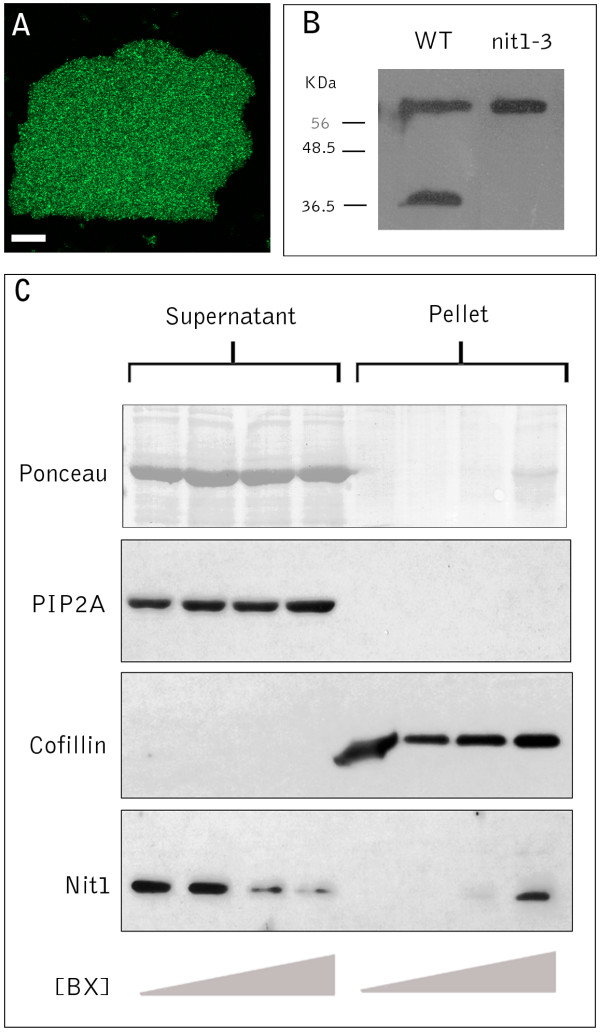
**Biochemical analysis of Nit1 aggregation**. (A) Extracts from bromoxynil-treated plants were made 4% in Triton X100 and centrifuged for 10 min at 10,000 g and the pellet was imaged by confocal microscopy. (B) Western blot of protein extracts from leaf tissue of wild type and the *Nit1-3 *mutant showing that the antibody recognizes the Nit1 protein in wild type but not the null mutant. (C) Aggregation of Nit1 into the low speed pellet fraction during herbicide-induced cell death. Three week old seedlings were imbibed in solutions of bromoxynil (0, 125, 250, 500 μM) for 1 h then extracted, adjusted to 4% Triton X100 and centrifuged at 10,000 g for 10 min. Proteins in the supernatant and pellet were separated by SDS-PAGE and various proteins detected by western blotting. Antibodies against PIP2A (plasma membrane intrinsic protein) and cofillin were used to show complete separation of the two fractions.

The extensive aggregation of GFP-Nit1 triggered by benzonitrile herbicides facilitated biochemical characterization of the process. GFP-NIT1 in unwounded plants is soluble and does not pellet during low-speed centrifugation (Fig 9). By contrast, we observed that the aggregates in GFP-Nit1 plants were stable during tissue disruption by sonication and partitioned into a low-speed Triton-X 100 insoluble pellet fraction by centrifugation (Fig. 9A). This low-speed pellet fraction was also found to contain the actin-binding protein cofillin, but not the membrane protein PIP2A, which resided in the low-speed supernatant; we therefore used these two proteins as markers for each fraction and as loading controls (Fig. 9B). The low-speed pelleting of Nit1 enabled us to test for herbicide-induced change in the solubility of nitrilase in wild type plants. A polyclonal antibody to recombinant Nit1 was produced and used to probe proteins isolated from the *nit1-3 *null mutant [38]. The crude serum recognizes two proteins, one of which is absent in the *nit1-3 *mutant (Fig. 9B), showing that the antibody faithfully recognizes Nit1 protein, the identity of the upper band is unknown and was not detected after affinity purification of the antibody against immobilized recombinant Nit1. Affinity-purified antibody was used to probe plants that were treated with bromoxynil under conditions that caused extensive aggregation in the GFP-Nit1 transgenic lines. In wild type plants treated with bromoxynil at concentrations that triggered aggregation of GFP-Nit1 in vivo, the Nit1 protein partitioned into the pellet fraction (Fig. 9C). Western blots of the GFP-Nit1 fusion protein showed comparable accumulation of the fusion protein in the low-speed pellet fraction (data not shown). These experiments provide an independent biochemical assay for a bromoxynil-induced change in solubility of Nit1 and suggest that the bromoxynil- and wound-induced aggregation of the GFP-Nit1 marker is not a fusion protein artifact.

## Discussion

**Figure 10:**
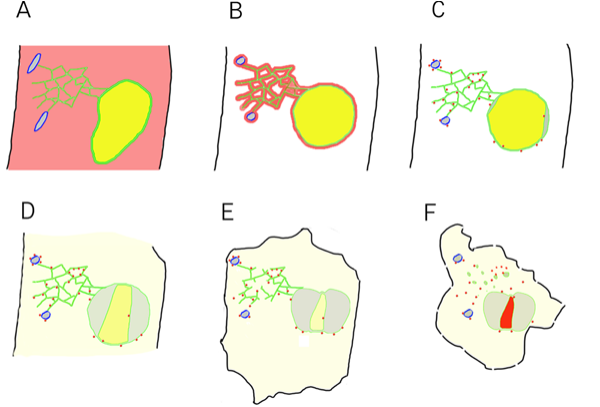
**Cytological events associated with wound-induced cell death**. (A) Unwounded cell. Fusiform bodies outlined in blue, cytoplasmic GFP-Nit1 in red, ER and nuclear envelope membranes shown in green, ER lumen contents shown in gray and nuclear lumenal contents shown in yellow, plasma membrane in black. (B – F) Post-wound events. (B)The earliest events documented are the collapse of fusiform bodies and nuclear hypertrophy. (C) Shortly after these early rapid events, aggregation of GFP-Nit1 occurs and the nucleus initiates contraction concurrent with separation of the nuclear envelope. (D) As contraction and lobing continue, contents of the nucleoplasm leak into the cytosol. It is currently unknown if this release is general or if there is specificity to the particular contents lost. (E) Later, after nuclear contraction has largely ceased, the tubular ER network that is still intact undergoes a dramatic loss of integrity and degenerates, forming vesicular structures. (F) This is followed later by intense staining of nuclei by propidium iodide and extensive cell shrinkage. Propidium iodide staining of nuclei is indicated in (F) by the red color of the nucleus.

This study was stimulated by the fortuitous observation that a GFP-Nit1 fusion protein rapidly underwent a change of state, from soluble to granular, in cells adjacent to a puncture wound. Because of technical difficulties associated with reproducibly producing puncture wounds, we surveyed a number of chemicals for their ability to induce GFP-Nit1 aggregation. We found that several benzonitrile herbicides caused effects that were similar to puncture wounds. Using a variety of GFP-based markers, we observed that cells abutting the wound site, or following treatment with benzonitriles, display a suite of dynamic morphological events that culminate in cellular contraction and cell death. Using an ER membrane marker we were able to simultaneously observe alterations in cortical ER tubules, fusiform bodies, the nuclear envelope and the nucleus. Similarly, using GFP-Nit1, we observed that the formation of nitrilase aggregates precedes nuclear and cellular contraction. Collectively, our data is consistent with a series of events that we have synthesized into a descriptive scheme (Fig. 10).

The earliest events after wounding were fusiform body alterations, aggregation of GFP-Nit1, and nuclear hypertrophy. Following herbicide treatment, we observed that fusiform body alterations preceded GFP-Nit1 aggregation (data not shown). Shortly after these early events, nuclear contraction ensues with a concomitant separation of the nuclear envelope and nucleoplasm leakage into the cytosol. It is currently unknown if this reflects a generalized loss of nucleoplasm or if there is specificity to the contents lost. Later, after nuclear contraction has largely ceased, the remaining tubular ER undergoes a dramatic loss of integrity and degenerates, forming vesicular structures. Intense staining of nuclei by propidium iodide and extensive cell shrinkage follows this. Since propidium iodide is a charged fluorophore that is largely precluded from entering vital cells, we interpret this late event to represent a major degeneration in the integrity of the plasma membrane and consider this the likely point of cell death, although this is an operational definition. Observations of a plasma-membrane marker suggest that the plasma membrane is intact at a gross level during the early stages of the wound response, although atypical involutions and extreme photo-sensitivity have been observed (data not shown).

The events we documented occur on a relatively rapid time scale. Typically, within minutes of wounding we observed detectable effects on the shapes of nuclei, fusiform bodies and the aggregation of GFP-Nit1. Within one to two hours of wounding, cells displayed intense staining of their contracted nuclei by propidium iodide. Although we did not obtain evidence for programmed cell death (PCD), this rapid response is compatible with evidence that plant cells undergo PCD by constitutively expressed molecular machinery [39]. It seems plausible that, by limiting possible pathogen access through a wound site, wound-induced cell death functions for the benefit of the organism as a whole.

Although we are far from a mechanistic picture of these events, our observations show that nuclear contraction can be uncoupled from cellular collapse. The known effects of benzonitrile herbicides on mitochondrial function may suggest that nuclear contraction is energy dependent. Since cellular collapse occurred after chloroxynil treatment, this may suggest that contraction is energy independent, perhaps unsurprising, since turgor pressure is generated in part through vacuolar ATPases and proton pumps.

Our focus in this study was on placing the aggregation of GFP-Nit1 in the context of cellular responses during wound-induced cell death. We could not identify any precedent in the literature for a similar transformation. We do not understand the mechanism by which GFP-Nit1 is converted from a soluble protein to a granular form. The observation that nitrilase 1 undergoes a change in sedimentation coefficient after chemically-induced injury in wild type plants suggests that the phenomenon is not an artifact of the GFP fusion protein. We do not know if nitrilase 1 is the only protein that exhibits such properties. However, we did not observe a similar response during the characterization of several hundred other GFP-protein fusions [24] (and unpublished results). The observation that the GFP-Nit1 aggregates have a relatively constant size that is roughly comparable to that of a secretory vesicle may suggest that the basis of aggregation is encapsulation or binding of nitrilase to a vesicular type of structure. However, the observation that treatment with Triton X100 does not disperse the aggregates indicates that they are not simple membrane-bound vesicles.

## Conclusion

Wounding and benzonitrile herbicides both induce rapid forms of cell death that yield collapsed cells with disrupted plasma membranes. One of the earliest responses in both of these cell death systems is the redistribution of Nit1 into aggregates that are visible in vivo as GFP-Nit1 granules or biochemically as a low-speed pelletable form of Nit1. Nit1 aggregation precedes nuclear contraction and cellular collapse, two classic cytological features of plant cell death. The use of GFP-Nit1 and other markers to image plant cell death in vivo has revealed novel subcellular responses that to our knowledge have not been previously described, such as nuclear envelope separation, formation of nuclear lobes and release of nuclear contents into the cytosol. Our data have enabled a new detailed descriptive model for plant cell death and raise new mechanistic questions.

## Methods

### Plant growth and preparation for microscopy

Plants were grown on agar-solidified media consisting of 0.5X MS salts (Gibco-BRL) and 0.8 % Agar (Research Organics). Prior to germination, seeds were chilled for 4 days on MS plates at 4°C then transferred to a growth cabinet and grown under continuous illumination at 300 μE m^-2^s^-1^. Unless otherwise stated, imaging experiments were performed on whole 4 – 8 day old seedlings mounted in 0.5X MS or an imaging buffer (IMB) composed of 0.5X MS salts, 25 μg/ml propidium iodide (Sigma) and 0.01% Silwet (Lehle Seeds, Tucson, AZ), which was added to facilitate propidium iodide penetration of the epidermal cuticle. To prevent specimen drift during time lapse experiments, whole seedlings in 0.5X MS were mounted by adding 2 volumes of molten 2% low melting point agarose (Research Organics), 2% high resolution 3:1 agarose (FMC) and 0.5X MS salts (Gibco-BRL) at 42°C. Cells showed active streaming after mounting, suggesting minimal stress induced by the mounting process.

### Targeted GFP lines

Most of the transgenic lines used for imaging in this paper have been described previously [24,37], but are described here for clarity. For GFP-cDNA lines, experiments were performed on T_3 _seed derived from the primary transgenic plants. Unless indicated otherwise, these lines contained a single GFP-cDNA fusion. The GFP fusions are available from the Arabidopsis Biological Resource Center (ABRC) at Ohio State University .

#### Nuclear markers

The N7 marker line (ABRC #CS84731) contains a fusion protein between GFP and the carboxy terminus of an ankyrin-repeat containing transcription-factor-like protein (Genbank Accession CAA16704). The N6 marker line (ABRC #CS84815) contains a near full-length fusion between an HMG-delta protein (Genbank accession Y14075) and GFP. In dividing cells it illuminates chromosomes aligned along the metaphase plate and thus likely associates with chromatin (26). In interphase cells, it illuminates the nucleoplasm. This line also contains a second PCR-detectable insert that contains an out-of-frame cDNA fusion to GFP.

#### ER markers

The mGFP5 line was generated by Wolf Scheible by transformation of the mGFP5 construct on plasmid pmGFP5-ER [37] into the Columbia ecotype of Arabidopsis. The Q4 ER membrane marker line (ABRC #CS84728) is a fusion between GFP and a novel protein with a predicted carboxy-terminal trans-membrane (Genbank Accession AAB71445).

#### Wound induced aggregates

The GFP-Nit1 marker comprises a full-length nitrilase 1 [38] (Genbank Accession U47114) fused to the C-terminus of GFP. Two lines were used in this study 35S-GFP-Nit1, in which the fusion protein is driven by the 35S promoter and, N1P2E, in which expression is driven by 1.8 kb of sequence upstream from the Nit1 start codon [30]. These two different lines possess similar expression levels in transgenic plants, however in comparison to 35S-GFP-Nit1, the N1P2E line shows reduced expression in root epidermal cells and guard cells

### Imaging

Agarose mounted seedlings were wounded by creating cuts through plant tissue with a razor blade or sharp forceps tips. Image data was collected after a brief (45 – 60 second) period of aligning the wound site with the field of view. During and prior to imaging experiments, agarose embedded specimens were covered with a humidifying dome to prevent desiccation. The majority of image series were obtained by collecting 20 – 25 μM deep Z-series (typically 15X 1.5 μm z-steps) for 30 – 60 time points at 120 sec intervals. 3-D time series were made from these data sets by making brightest-point reconstructions of each Z-series using the BioRad software package LaserSharp (BioRad, Hercules, CA). For some time series, reconstructions were performed manually in NIH image using brightest point reconstructions (Wayne Rasband, RSB, NIH, Bethesda Maryland). A Nikon inverted fluorescence microscope equipped with a Nikon 60X 1.2 numerical aperture water immersion objective and a Bio-Rad MRC 1024 confocal head with a krypton-argon laser. EGFP was excited at 488 nm and emitted fluorescence was collected through a 525-30 band pass filter. Chloroplast autofluorescence and propidium iodide fluorescence were obtained by excitation at 568 nm and collecting emitted fluorescence through a 596 – 615 nm band pass filter.

### Chemical treatments

Chloroxynil has a higher solubility in ethanol and water than bromoxynil and was used in imaging experiments. Both herbicides show similar toxicity to Arabidopsis using a germination assay [30]. A 1 M stock of Chloroxynil (ChemServices, West Chester, PA) was made in ethanol. For imaging experiments, a 1 mM solution of Chloroxynil in IMB was made and 100 μl of this solution was overlaid onto to seedlings mounted in 100 μl of agarose, yielding a final concentration of 500 μM.

### Nit1 antibody production

Recombinant Nit1 was produced in *E. coli *as a His-tagged thioredoxin fusion protein using the vector pET32(A) (Novagen, Madison, WI) and purified by chromatography on a nickel-chelate resin, (Novagen, Madison, WI). Polyclonal serum against recombinant Nit1 protein was made by a commercial provider (Cocalico, Reamstown, PA). The Nit1 antibodies were affinity purified against 2 mg of purified recombinant Nit1 adsorbed onto a 6 × 8 cm piece of nitrocellulose membrane and eluted off the membrane by treatment with 200 mM glycine, 150 mM NaCl pH 2.0. The eluted antibodies were neutralized to pH 7.0 with Tris base, dialysed overnight against Tris-buffered saline and concentrated to 2 mg/ml using a spin column concentrator with a 15 kDa cutoff, the concentrated antibody was diluted to 1 mg/ml with glycerol.

### Nit1 pelleting assays

Herbicide-treated plants were transferred into a 3X-weight volume of ice-cold granule isolation buffer (GEB, 400 mM sucrose, 75 mM KCl, 50 mM PIPES pH 6.9, 10 mM MgCl_2_, 1 mM EGTA, 1 mM DTT, 1 mM PMSF, 1 μg/ml leupeptin, 1 μg/ml aprotinin). They were then sonicated, filtered through Miracloth and Triton-X 100 was added to a final concentration of 4%. After 10 min incubation on ice, the extract was centrifuged at 10,000 g for 10 min. The supernatant was removed and retained and the pellet was washed by with GEB + 4% Triton-X 100. Protein from the supernatant fraction was precipitated using a chloroform-methanol extraction protocol [40]. The pellet fraction and precipitated low-speed supernatant fractions were resuspended in equal volumes of SDS-PAGE sample buffer, typically 500 μl per 100 mg input tissue (fresh weight).

For herbicide induced-pelleting, 2–3 week old Arabidopsis seedlings were transferred into a solution of ddH_2_0, 0.01% Silwet and varying doses of Bromoxynil. After 1 h incubation at room temperature with gentle shaking, seedlings were transferred to an eppendorf tube, placed on ice and disrupted by sonication in GEB as described above.

### Protein analysis

SDS-PAGE was performed using 8 – 20 % gradient mini-gels, prepared and run as described in Ausubel et al. [41]. For western analysis, proteins were transferred onto nitrocellulose membranes using a semi-dry electroblotter in Towbin transfer buffer [42]. Westerns were developed using Pierce SuperSignal HRP substrate (Pierce, Rockville, IL). The cofillin and Pip2A used as control antibodies were obtained from Rose Biotechnology [44] and used at 1/2000 dilutions. The Nit1 antibodies were used at 1/1000 dilution.

## Supplementary Material

Additional File 1 **Wound induced granulation of GFP-Nit1 demonstrating action at a distance **A 35S GFP-Nit1 root was wounded above the root meristem with a syringe tip, quickly aligned for microscopy and then imaged at a single focal plane at 4 second intervals for 8 minutes (the wound site is out of the focal plane of the time series, but is visible as the dark area of separated cells in the center of the root tip). Initially, the GFP-Nit1marker is visible as diffuse fluorescence. Over the course of several minutes, bright granular structures become evident concomitant with a decrease in cytoplasmic fluorescence, suggesting a redistribution of the marker into granules. The total time of the image series is 8 minutes. In other time series which have imaged the process over a longer time period, we have observed reversion of the marker to cytoplasmic distribution with resumption of cytoplasmic streaming (see additional file3). The action-at-a distance observed with this marker suggests that the marker is responsive to a motile signal is sent after wounding. The data shown in figure1A were derived from this data set. Click here for file

Additional File 2 **Collapse of cells within the wound zone – wound induced granulation of GFP-Nit1 followed by cellular contraction and detachment from neighboring cells **The cotyledons of agarose imbedded GFP-Nit1 lines (N1P2E) were wounded with razor incisions and subsequently imaged by collecting confocal Z-series of a region abutting the wound at 60 second intervals. (2A) Shown is a detail of a time-series made from brightest point 3-dimensional reconstructions of the acquired Z-series. Immediately after wounding, the center cell shown in the image series displayed GFP-Nit1 granules. After a short period of time, the cell started contracting with a extensive detachment of the cell from its cell walls. (2B) The full frame image data that Movie 2A was derived from. Note the numerous cells in the wound zone displaying granulation after wounding and their subsequent contraction over the course of the time series. Figure 2B in the manuscript was derived from this data set (see additional file 8). Click here for file

Additional File 3 **Reversion of GFP-Nit1 granulation during the transmitted response **An agarose mounted 35S GFP-Nit1 plant was wounded above the root meristem and imaged by the acquisition of Z-series at 2 minute intervals for 90 minutes. The wound site was above the field of view. By the time of acquiring the first time point, the granulation response had spread throughout much of the root meristem. Over time, the granules revert back to a cytoplasmic distribution pattern and the cells resume cytoplasmic streaming. Images are brightest point reconstructions of Z-series collected at 2 minutes intervals after wounding. Click here for file

Additional File 4 **Nuclear contraction during wound induced cell death **The hypocotyl of an agarose imbedded nuclear marker line (N6) was wounded with a razor blade and them imaged at 2 minute intervals by collecting confocal Z-series. Propidium iodide was included in the imaging buffer to monitor plasma membrane integrity. Following wounding,, the nucleus on the left displays hypertrophy relative to the normal lentoid shape of unwounded cells (see the nuclei in cells on the right hand side in the movie). Over the course of the time series, the nucleus contracts concomitant with a decrease in GFP fluorescence, most evident in the time series between the 16 and 20 minute time points shown. This is followed later by intense staining of the contracted nucleus by propidium iodide. Click here for file

Additional File 5 **Degeneration of the cortical ER network during wound induced cell death **A wound-proximal petiole epidermal cell of the ER membrane marker line Q4 was imaged. Part of the ER network formed bubble-like structures rapidly following wounding, and the remaining intact tubular ER then degenerates, forming large numbers of vesicular structures. Images are brightest point reconstructions of Z-series collected at 2 minutes intervals after wounding. Click here for file

Additional File 6 **Chloroxynil induced cell death – extensive cellular collapse without nuclear contraction **The nuclear marker line N7 was mounted in agarose, and chloroxynil was added (final concentration 500 μM) and imaging was initiated collecting Z-series of the hypocotyl-root junction at 2 minute intervals. Loss of nuclear label into the cytoplasm and a generalized loss of GFP fluorescence were followed by staining of nuclei by propidium iodide and massive cellular collapse. The shape of the nuclei remain constant throughout the experiment and do not display the characteristic contractions seen during wound induced cell death.. Click here for file

Additional File 7 **Fusiform body alterations during Chloroxynil triggered cell death **Epidermal hypocotyl cells of an agarose mounted Q4 seedling were imaged by acquiring z-series at 120 second intervals. After the three z-series had been acquired, chloroxynil was added to a final concentration of 500 μM. Active streaming is evident during the first 3 time points. By 4 minutes after herbicide application, streaming ceases, evident by the lack of movement of fusiform bodies and the tubular ER network, which are usually highly dynamic. Shortly thereafter, fusiform bodies transform into spherical structures, which also occurs during wound-induced cell death. The data shown in figure 8 were derived from this data set. Click here for file

Additional File 8 **Collapse of cells within the wound zone – wound induced granulation of GFP-Nit1 followed by cellular contraction and detachment from neighboring cells. **The full frame image data that Movie 2A was derived from. Note the numerous cells in the wound zone displaying granulation after wounding and their subsequent contraction over the course of the time series. Figure 2B in the manuscript was derived from this data set. Click here for file
